# Fibromatosis (desmoid tumor) of the breast mimicking a case of ipsilateral metachronous breast cancer

**DOI:** 10.1186/1477-7819-4-57

**Published:** 2006-08-22

**Authors:** Stephen P Povoski, Rafael E Jimenez

**Affiliations:** 1 Division of Surgical Oncology, Department of Surgery, The Arthur G. James Cancer Hospital and Richard J. Solove Research Institute and Comprehensive Cancer Center, The Ohio State University, Columbus, Ohio, 43210, USA; 2 Department of Pathology, The Arthur G. James Cancer Hospital and Richard J. Solove Research Institute and Comprehensive Cancer Center, The Ohio State University, Columbus, Ohio, 43210, USA

## Abstract

**Background:**

Fibromatosis or desmoid tumor of the breast is an extremely rare entity. While it lacks a metastatic potential, it can grow aggressively in a locally infiltrating pattern. The failure to recognize this as a finite entity within the breast can lead to local recurrence after incomplete excision.

**Case presentation:**

We report a case of a 70 year old patient with a remote history of invasive breast cancer (treated twelve years earlier by lumpectomy, axillary lymph node dissection, postoperative radiation therapy, and five years of tamoxifen) who developed fibromatosis within another quadrant of the same breast that clinically, mammographically, and sonographically mimicked that of the development of an ipsilateral metachronous breast cancer. After the initial diagnosis of fibromatosis was made on a minimally invasive ultrasound guided biopsy, it was successfully treated by wide local excision.

**Conclusion:**

After appropriate recognition, wide local excision can be the appropriate surgical management strategy for fibromatosis of the breast.

## Background

Fibromatosis or desmoid tumor of the breast is an extremely rare entity. Multiple case reports have been published [1-22], as well as a few comprehensive series [23-27]. Some have suggested that this entity arises *de novo *from within the breast parenchyma itself [13,23,24], while others have suggested that it arises *de novo *from the aponeurosis overlying the pectoralis major muscle [13,24,28]. Still others contend that it may arise secondary to a previous history of breast surgery or trauma [5,6,25,28]. Despite its lack of metastatic potential, fibromatosis can grow aggressively in a locally infiltrating pattern [24,29-31]. The failure to recognize this process as a finite entity within the breast and the temptation to discount it as "scar tissue" from a previous breast biopsy or trauma may ultimately lead to local recurrence within the breast if inadequately treated [22].

This case report describes a patient with a remote history of breast cancer that was treated twelve years earlier by lumpectomy, axillary lymph node dissection, postoperative radiation therapy, and five years of tamoxifen who developed a case of fibromatosis within another quadrant of the same breast that clinically, mammographically, and sonographically mimicked that of an ipsilateral metachronous breast cancer.

## Case presentation

The patient is a 70 year-old white female who has the previous history of a right breast cancer that was treated approximately twelve years ago by a lumpectomy to the upper central portion of her right breast, right axillary lymph node dissection, and postoperative radiation therapy for a pT1c, pN0, M0, estrogen receptor positive, and progesterone receptor positive invasive ductal carcinoma. She was maintained on tamoxifen for five years thereafter. In her routine annual follow-up at The JamesCare Breast Center of The Arthur G. James Cancer Hospital, she reported feeling a new pea-sized palpable event in the inferolateral aspect of her right breast for only one-week duration.

On clinical breast examination, the patient was noted to have a well-healed periareolar, curvilinear scar along the superior aspect of her right breast from approximately the 10 o'clock to 2 o'clock axis. There were no associated palpable events within this region of her right breast representing the area of her previously treated breast cancer. However, within the mid to peripheral portion of the lower outer quadrant of her right breast in the 7 o'clock axis, the patient had a less than one centimeter suspicious palpable nodule that appeared to be relatively superficially located within the breast tissue.

**Figure 1:**
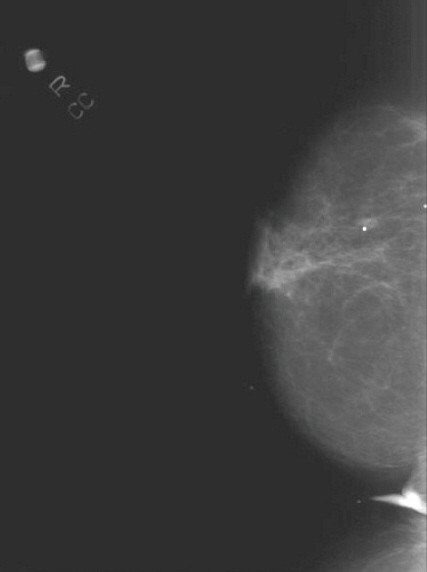
Cranial-caudal mammographic view of the right breast. The corresponding mammographic density is marked by a radiopaque BB in the mid-portion of the lateral pole of the breast.

**Figure 2:**
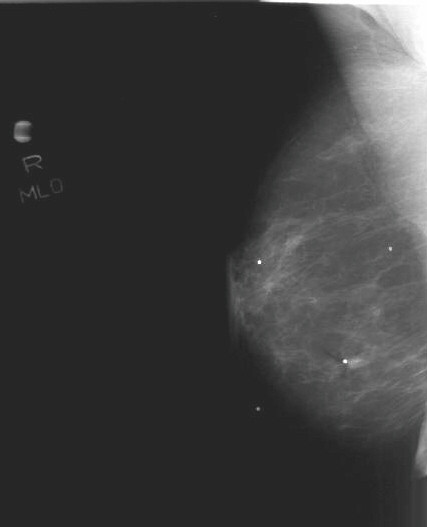
Medial-lateral oblique mammographic view of the right breast. The corresponding mammographic density is marked by a radiopaque BB in the mid-portion of the lower pole of the breast.

**Figure 3:**
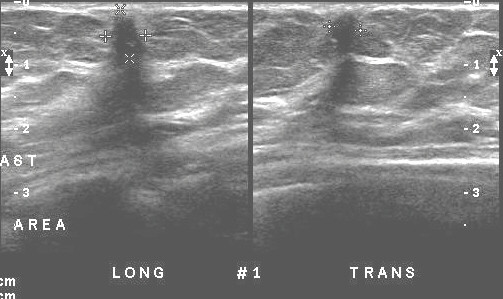
Ultrasound of the right breast.

Mammography (Figure 1 and 2) showed stable, asymmetrical, increased density to the tissue within the upper retroareolar portion of her right breast, consistent with her prior lumpectomy site. However, a new 0.8 × 0.5 cm mammographic density was noted in the inferolateral portion of her right breast. Targeted right breast ultrasound (Figure 3) showed a 0.78 × 0.63 × 0.49 cm hypoechoic lesion that was located in the 7 o'clock axis and approximately 4 cm from the nipple, which was associated with acoustic shadowing. This was thought to be suspicious for malignancy and was classified as BI-RADS 4, for which a biopsy was recommended.

**Figure 4:**
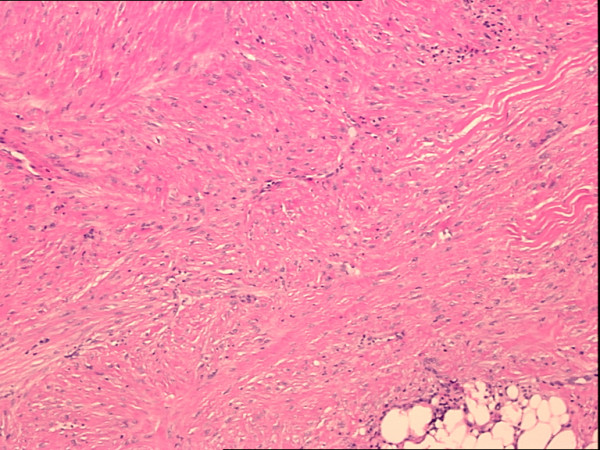
Low power (40×) H&E view showing the presence of long interlacing fascicles of bland appearing spindle cells with a moderate amount of collagen deposition and focal hyalinization.

**Figure 5:**
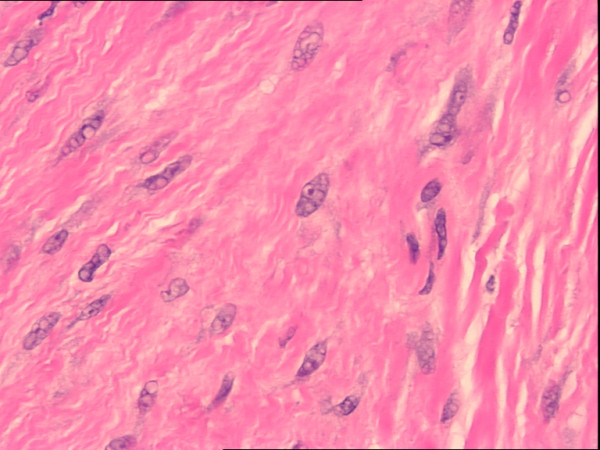
High power (400×) H&E view showing spindle cells with classic myofibroblastic features, consisting of vesicular nuclei and small nucleoli, indistinct cytoplasm, and interstitial collagen.

A minimally invasive right breast ultrasound-guided 8 gauge Mammotome^® ^vacuum-assisted biopsy was performed. Microscopic evaluation revealed fragments of breast parenchyma in which a bland spindle cell proliferation was present, forming long interlacing fascicles (Figure 4). The cells showed cigar-shaped nuclei with vesicular chromatin and pinpoint nucleoli that were dispersed amidst varying degrees of collagen deposition, ranging from scant to thick bundles (Figure 5). In some fragments, the proliferation could be seen entrapping lobular units. Immunohistochemical stains for cytokeratin were negative.

The patient was subsequently taken to the operating room at to The Arthur G. James Cancer Hospital and underwent an ultrasound directed wide local excision of the inferior portion of her right breast in the 7 o'clock axis within the mid to peripheral breast field.

**Figure 6:**
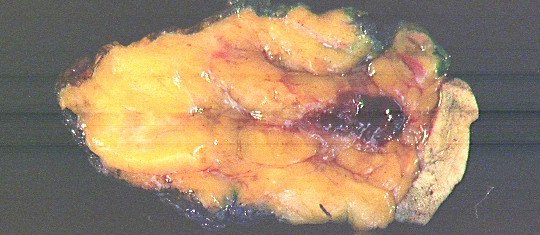
Gross cross-sectional view of pathology specimen. The residual area of fibromatosis is noted along the inferior aspect of the hemorrhagic Mammotome^® ^biopsy cavity.

Gross pathologic evaluation of the specimen, which overall measured 7.5 × 4.5 × 4.0 cm in size, revealed a 0.8 × 0.7 × 0.4 cm gray-white firm mass abutting a 2.5 × 2.0 × 1.2 cm biopsy cavity (Figure 6). Microscopic evaluation revealed residual fibromatosis. All surgical margins were negative. The residual fibromatosis came to within 0.6 cm of the overlying skin margin and was 1.1 cm from the closest parenchymal margin. No evidence of carcinoma was seen.

The patient is currently nine months out from her wide local excision of fibromatosis of her right breast and she remains disease free.

## Discussion

While Lopez *et al *[18] previously reported a very unique case of fibromatosis (desmoid tumor) mimicking recurrent breast cancer within the lumpectomy bed of a patient treated two years earlier for invasive breast cancer, our current patient represents a similarly inimitable case of fibromatosis arising from within a separate quadrant of the same breast of a patient successfully treated for ipsilateral invasive ductal carcinoma some twelve years earlier, and thus mimicking an ipsilateral metachronous breast cancer. Clearly, these two unique case reports both highlight the manner in which fibromatosis can clinically, mammographically, and sonographically mimic the presentation of any breast cancer.

Since fibromatosis of the breast is such a rarely reported event in the literature, no clear-cut guidelines have been established to define what exact standards should be applied to the definition of a complete wide local excision. While incomplete excision with positive surgical margins has not always been associated with recurrence in some selected patients [23,25], negative surgical margins in most patients have generally been associated with decreased likelihood of local recurrence [24]. As with the surgical management of breast cancer or phyllodes tumors, we tend to all strive for the "ideal" one-centimeter surgical margin. In this regard, such a goal of one centimeter seems a reasonable aim in the "ideal" surgical world.

Thus, there seems to be generalized agreement that early recognition and appropriate complete wide local excision of fibromatosis confined to the breast alone is the appropriate first-line treatment of choice. Such a local surgical management strategy will ultimately prove to be curative in most cases. This strategy, embraced by the present authors, is highly dependent on the appropriate recognition of this entity in pre-excisional diagnostic biopsies, such as needle core or Mammotome^® ^vacuum-assisted biopsies. Failure to recognize fibromatosis as a finite entity within the breast and the temptation to discount this as "scar tissue" from a previous breast biopsy or from previous breast trauma may potentially lead to local recurrence of fibromatosis within the breast. This failure can ultimately result in the need for more radical resections to obtain clearance of the surgical margins [22,32,33]. Avoidance of the need of such a more radical approach is clearly advantageous to the patient from a cosmetic and functional standpoint [13,17,21-23]. In the current case, clinico-pathologic correlation was instrumental in avoiding such a scenario, since relevant clinical information (i.e., recent onset of the lesion, location of the lesion away from the previous surgical site) was accurately conveyed to the pathologist and prompted the diagnosis of fibromatosis. Close communication between the surgeon and the pathologist is fundamental to the appropriate surgical management of this lesion.

## Competing interests

The author(s) declare that they have no competing interests.

## Authors' contributions

**SPP **was the operating surgical oncologist. He was the principle investigator who prepared, organized, wrote, and edited all aspects of the manuscript.

**REJ **performed the gross and microscopic pathologic evaluation of the pathology specimen. He prepared all of the histology figures in the manuscript. He read, edited, and approved the final version of the manuscript.
